# Trimethylamine N-oxide and its precursors in relation to blood pressure: A mendelian randomization study

**DOI:** 10.3389/fcvm.2022.922441

**Published:** 2022-07-22

**Authors:** Han Wang, Qiang Luo, Xunshi Ding, Lifang Chen, Zheng Zhang

**Affiliations:** Department of Cardiology, The Third People's Hospital of Chengdu, Affiliated Hospital of Southwest Jiaotong University, Chengdu, China

**Keywords:** trimethylamine N-oxide, carnitine, blood pressure, Mendelian randomization, Genome-Wide Association Studies

## Abstract

**Objective:**

Previous studies have demonstrated that trimethylamine N-oxide (TMAO) and its precursors, including choline, betaine, and carnitine, are closely associated with blood pressure (BP) changes. Nevertheless, with the limitation of reverse causality and confounder in observational studies, such a relationship remains unclear. We aimed to assess the causal relationship of TMAO and its precursors with BP by the Mendelian Randomization (MR) approach.

**Method:**

In this study, two-sample MR was used to reveal the causal effect of TMAO and its precursors on BP. Pooled data of TMAO and its precursors was from genome-wide association studies (GWAS) which includes summary data of human metabolome in 2,076 European participants from Framingham Heart Study. Summary-level data for BP was extracted from the International Consortium of Blood Pressure-Genome Wide Association Studies. Inverse variance weighted (IVW), MR Egger regression, Maximum likelihood, Weighted median, and MR pleiotropy residual sum and outlier test (MR-PRESSO) were used in this MR analysis.

**Results:**

A total of 160 independent SNP loci were associated with TMAO and three precursors, including 58 associated with TMAO, 29 associated with choline, 44 associated with betaine, and 29 associated with carnitine, were selected. MR results suggested that a 1 unit increase in TMAO should be associated with a 1SD increase in systolic BP mmHg (beta: 0.039, SE, 0.072, *p* = 0.020). Additionally, our findings also indicated that a 1 unit increase in carnitine should be associated with a 1SD increase in systolic BP mmHg (beta: 0.055, SE: 0.075, *p* = 0.039). This result was also confirmed by sensitivity analysis methods such as Maximum likelihood, MR-PRESSO, and Weighted median. No effects of betaine or choline on systolic or diastolic BP were observed in the present study.

**Conclusion:**

Our study provides evidence of a causal relationship of TMAO and its precursors with BP, suggesting that mediating the generation of TMAO would be beneficial for lowering BP.

## Introduction

Hypertension is the most common chronic disease and an independent risk factor for cardiovascular diseases including heart failure, coronary heart disease, and myocardial infarction. Accumulating evidence has indicated that for every 5 mmHg increase in systolic blood pressure (BP), the risk of myocardial infarction rises by 5% and mortality by 10% (1). Despite recent advances in the pathogenesis and therapeutic agents, hypertension, as a global public problem, remains the leading cause of premature death and disability (2). At present, it is generally accepted that the pathogenesis of hypertension mainly involves the sympathetic nervous system and peripheral vascular remodeling, including the renin-angiotensin-aldosterone system (RAAS), immune system, calcium channels, and endothelial dysfunction (3). Several well-known risk factors such as hyperlipidemia and diabetes contribute to some degree to hypertension. However, many risk factors for hypertension remain unknown.

Recently, the interaction between the gut microbiota and the host has attracted significant attention. Numerous studies have reported that gut flora are involved in the pathogenesis of many diseases, especially in cardiovascular disease (4). More importantly, some metabolites of gut microbes can also be absorbed into the circulatory system, affecting the host and disease. Among these metabolites, one of the most essential bioactive molecules is trimethylamine N-oxide (TMAO), which is derived from foods rich in choline, phosphatidylcholine, and betaine (e.g., red meat, eggs, milk, etc.) (5). Previous studies have demonstrated that TMAO is important for the development and progression of cardiovascular diseases (e.g., hypertension, atherosclerosis, coronary artery disease, and heart failure) (4–6). Several animal experiments have found that infusion of TMAO can prolong the hypertensive effects in rats, and an increase in TMAO levels in spontaneously hypertensive rats leads to higher plasma osmolality, causes water reabsorption, and ultimately results in an elevated BP (7). Subsequent systematic review with 8 cohort studies containing 6,176 hypertensive subjects and 11,750 controls found that high concentrations of TMAO were associated with a higher prevalence of hypertension compared with low TMAO concentrations (RR: 1.12; 95% CI: 1.06, 1.17; *P* < 0.0001) (8). These findings suggested a direct relationship between TMAO and BP. In addition, an immediate effect of TMAO's precursors including choline, betaine, and carnitine on BP has also been observed in several previous reports (9–11), and in particular, these precursors are thought to regulate BP in humans (12). However, these current results are somewhat conflicting. For example, some studies argue that these molecules can lower BP, but others have yielded opposite results (10, 11).

These observational studies addressed the association of TMAO and its precursors with BP, but the findings are often controversial due to the potential confounders and reverse causation in epidemiological studies. Randomized controlled trials (RCTs) are the most reliable way to make causal inferences in epidemiological studies. Nevertheless, it is often not easy to directly investigate the etiology of disease because of the difficulty of design and implementation in RCT and the medical ethical issues that must be considered. Mendelian randomization (MR) studies infer the causality of exposure factor on the outcome using genetic variants that have strong associations with the exposure factor as instrumental variables (IVs) (13). According to Mendelian inheritance laws, parental alleles are randomly assigned to offspring, similar to the randomization process in RCTs; furthermore, because genetic variation is innate, the association of genetic variation with the outcome is consistent with a causal timing, and is not influenced by acquired factors (13). Therefore, MR can effectively overcome bias caused by confounding and reverse causality. Therefore, a 2-sample MR approach was used to investigate the effects of TMAO and its precursors including choline, carnitine, and betaine on BP.

## Population and method

### Study design

This study analyzed the causal effect of TMAO and its precursors on BP using a 2-sample MR analysis. TMAO and its precursors were used as exposure factors, and BP was used as an outcome indicator in our analysis.

### Data source

#### TMAO and its precursors

Summary data for TMAO and its precursors, including betaine, carnitine, and choline, were obtained from a GWAS of human metabolome that included 2,076 European participants from the Framingham Heart Study (14). Considering that gene loci of these metabolites rarely reach genome-wide significance in GWAS, single nucleotide polymorphisms (SNPs) with suggestive genome-wide significance thresholds (i.e., *P* < 5 × 10^−5^) were selected as IVs in this study (Table 1).

**Table 1:** Gut metabolites and hypertension summary data sources.

**Trait**	**Year**	**Sample**	**Case**	**Control**	**Population**
Trimethylamine N-oxide	2013	2,076	NA	NA	Europe
Betaine,	2013	2,076	NA	NA	Europe
Carnitine	2013	2,076	NA	NA	Europe
Choline	2013	2,076	NA	NA	Europe
Systolic blood pressure	2018	757,601	NA	NA	Europe
Diastolic blood pressure	2018	757,601	NA	NA	Europe

#### Blood pressure

Summary data for International Consortium of Blood Pressure Genome-Wide Association Studies were obtained from a GWAS meta-analysis including about 200,000 European cases, with adjustments for sex, age, body mass index, within cohort stratification, and antihypertensive medication use (15). Details of the included cohorts were described in the meta-analysis (Table 1).

### Instrumental variables selection

**Figure 1:**
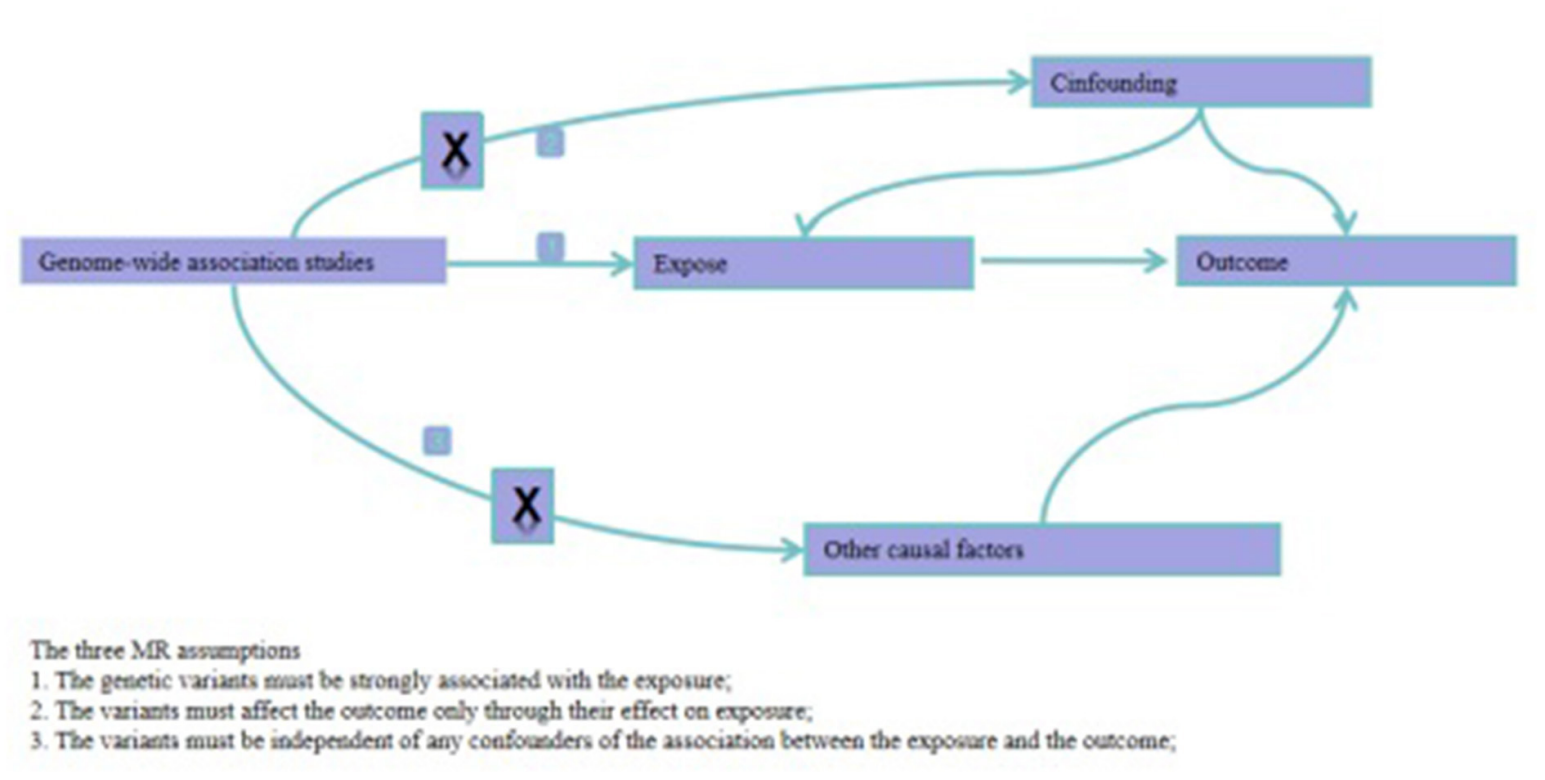
Directed acyclic graphs for the classical Mendelian randomization designs. The arrows denote causal relations between two variables, pointing from the cause to the effect. The causal pathway is blocked if “X” is placed in the arrowed line. MR, Mendelian randomization.

IVs were selected based on the following three criteria: (1) IVs were significantly associated with TMAO and its precursors: (2) IVs were not associated with BP; (3) Only TMAO and its precursors affected BP. Only SNPs that simultaneously met the above criteria were included as IVs (Figure 1).

We extracted SNPs from the gut metabolite database using Plink software with the following criteria: (1) *P* < 5 ^*^ 10^−5^; (2) Linkage disequilibrium parameters *r*^2^ < 0.01; (3) Gene distances = 10,000 kb. Furthermore, TMAO and its precursors could not be indirectly linked to BP *via* confounding factors in the MR; (4) SNPs with incompatible alleles and palindromic SNPs with minor allele frequencies close to 0.5 were removed from the analysis.

Based on the above principles, we utilized Catalog and Phenoscanner to explore whether the included SNPs were associated with confounders and, if not associated, the SNP was included (high salt intake, alcohol intake, obesity and low physical activity) (16). Further, we also used the F-statistic values to determine the strength of correlations of IVs with TMAO and its precursors to avoid bias from weak IVs (17). When the F-statistic value is >10, it is generally considered that there is no bias of weak IVs. Relevant information for each SNP as IVs, including major alleles, allele frequencies, beta coefficients, *P*-values, and standard errors, were collected.

## MR analysis

### Effect size estimates

This study mainly used the inverse variance weighted (IVW) method to evaluate the relationship of TMAO and precursors with systolic and diastolic BP. The outcomes of IVW were the major indicators of this study, which used random-effects model if there was heterogeneity; otherwise, the fixed effects model (18). In addition, we also used MR-Egger, Maximum likelihood, Weighted median, and MR-PRESSO for additional analysis to further validate the reliability of the IVW results (19–22).

### Assessment of horizontal pleiotropy and heterogeneity

First, we used the MR-PRESSO method to detect outliers and reanalyze them after removing them (22). The “leave-one-out” sensitivity analysis was performed by IVW method of the remaining SNPs after excluding 1 SNP each time, thereby assessing the effect of the individual SNPs on systolic and diastolic BP. In addition, MR- Egger was also used to test the horizontal pleiotropy for IVs, and the effect of genetic pleiotropy was considered to be small if the intercept of the MR-egger regression line was close to zero (23). Meanwhile, Cochran's Q was also used to examine the heterogeneity of IVs (24).

Statistical analysis was performed using the TwoSampleMR package in R software, and all results were expressed as OR and its 95% CI, with a *P*-value < 0.05 being a statistically significant difference.

## Results

### SNP selection

Included SNP locus information is provided in the Supplementary Table. In this study, rs16840689 (betaine), rs2074755 (betaine), rs1704040 (TMAO), rs8090864 (TMAO), rs4884119 (TMAO) were found to be closely related to confounding factors, so they were excluded. Overall, we selected a total of 151 independent SNP loci associated with TMAO and three precursors, including 51 associated with TMAO, 29 associated with choline, 42 associated with betaine, and 29 associated with carnitine. All had F-values >18.77, suggesting a low probability of the presence of weak IVs.

### MR statistical results

**Figure 2:**
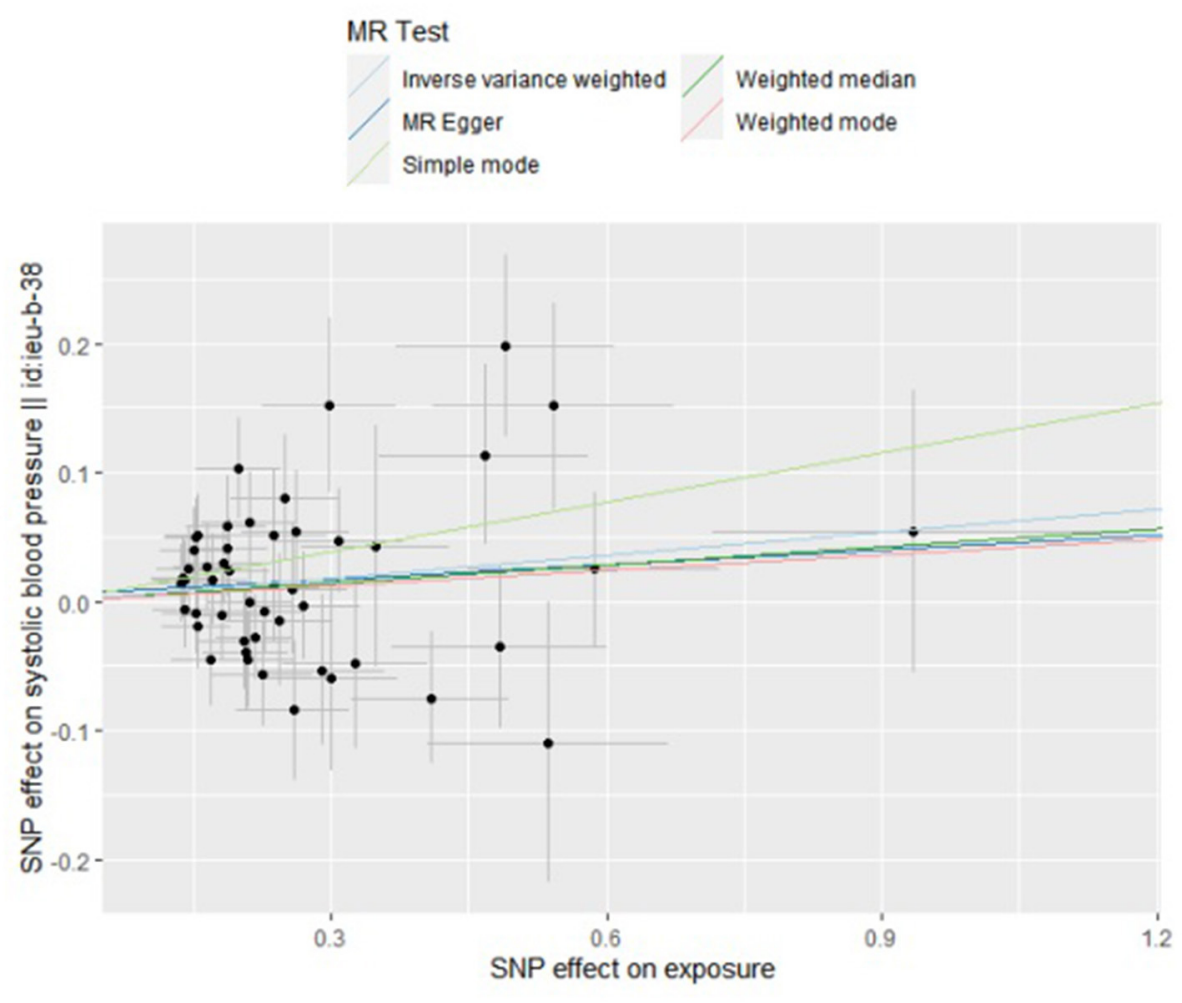
Scatter plot to visualize causal effect of trimethylamine N-oxide on the risk of systolic blood pressure in pregnancy. The slope of the straight line indicates the magnitude of the causal association. IVW indicates inverse-variance weighted; and MR, Mendelian randomization.

**Figure 3:**
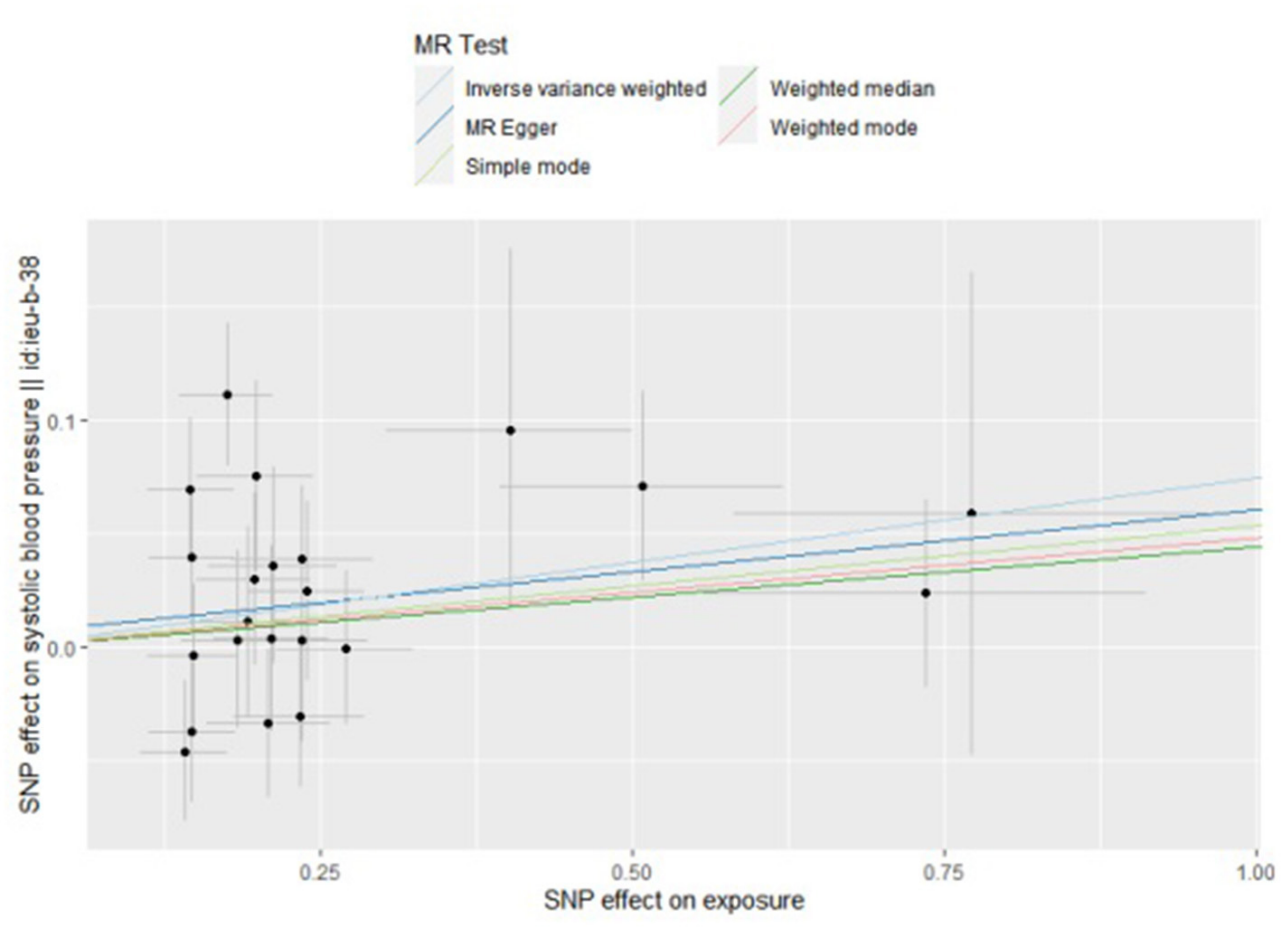
Scatter plot to visualize causal effect of carnitine on the risk of systolic blood pressure in pregnancy. The slope of the straight line indicates the magnitude of the causal association. IVW, inverse-variance weighted; and MR, Mendelian randomization.

By the IVW method, we observed that TMAO and carnitine were associated with systolic rather than diastolic BP. Considering the presence of heterogeneity, we used a random-effects model for IVW, whose results suggested that a 1 unit increase in TMAO should be associated with a 1 SD increase in systolic BP mmHg (beta: 0.055, SE: 0.075, *p* = 0.039) (Table 2; Figure 2). This result was also confirmed by sensitivity analysis methods such as Maximum likelihood (beta: 0.062, SE: 0.027, *p* = 0.020), MR-PRESSO (beta: 0.060, SE: 0.029, *p* = 0.042). Additionally, our findings also indicated that a 1 unit increase in carnitine should be associated with a 1 SD increase in systolic BP mmHg (beta: 0.075, SE: 0.036, *p* = 0.039) (Table 2; Figure 3). This result was confirmed by Maximum likelihood (beta: 0.077, SE: 0.032, *p* = 0.017) (Table 2). No effects of betaine or choline on systolic or diastolic BP were observed in the present study.

**Table 2:** MR analysis of trimethylamine N-oxide and its precursors on blood pressure.

**Outcome**	**Exposure**	**Nsnp**	**MR Egger**		**Inverse**		**Maximum**		**Weighted**		**MRPRESSO**		**Heterogeneity**		**Pleiotropy**	
					**variance weighted**		**likelihood**		**median**							
			**Beta ±SE**	***P-*value**	**Beta ±SE**	***P-*value**	**Beta ±SE**	***P-*value**	**Beta ±SE**	***P-*value**	**Beta ±SE**	***P-*value**	***P-*value**		***P-*value**
Systolic blood pressure	Trimethylamine_ N_oxide	49	0.039 ± 0.072	0.594	0.060 ± 0.029	0.036	0.062 ± 0.027	0.020	0.046 ± 0.040	0.245	0.060 ± 0.029	0.042	0.122		0.751
Systolic blood pressure	Carnitine	27	0.055 ± 0.075	0.472	0.075 ± 0.036	0.039	0.077 ± 0.032	0.017	0.044 ± 0.048	0.358	0.075 ± 0.036	0.052	0.090		0.765
Systolic blood pressure	Choline	28	−0.053 ± 0.066	0.425	−0.033 ± 0.040	0.413	−0.033 ± 0.029	0.251	−0.057 ± 0.043	0.184	−0.018 ± 0.034	0.608	0.002		0.694
Systolic blood pressure	Betaine	42	0.055 ± 0.054	0.310	−0.005 ± 0.033	0.890	−0.005 ± 0.024	0.845	0.022 ± 0.037	0.559	−0.004 ± 0.027	0.876	0.001		0.172
Diastolic blood pressure	Trimethylamine_ N_oxide	51	−0.019 ± 0.041	0.640	0.001 ± 0.018	0.993	0.001 ± 0.014	0.991	0.013 ± 0.022	0.562	0.001 ± 0.018	0.993	0.001		0.600
Diastolic blood pressure	Betaine	42	0.018 ± 0.031	0.566	−0.003 ± 0.019	0.855	−0.004 ± 0.014	0.788	0.020 ± 0.021	0.351	0.004 ± 0.015	0.787	0.001		0.393
Diastolic blood pressure	Carnitine	26	0.001 ± 0.034	0.992	0.003 ± 0.016	0.841	0.003 ± 0.016	0.835	0.022 ± 0.025	0.377	0.003 ± 0.016	0.843	0.378		0.904
Diastolic blood pressure	Choline	28	−0.006 ± 0.044	0.888	−0.009 ± 0.027	0.728	−0.010 ± 0.017	0.548	−0.008 ± 0.026	0.774	0.001 ± 0.024	0.993	0.001		0.931

MR-PRESSO, MR pleiotropy residual sum and outlier te.

### Horizontal pleiotropy and heterogeneity

The results of horizontal pleiotropy and heterogeneity suggested the absence of horizontal pleiotropy but heterogeneity (Table 2). Considering the existence of heterogeneity, a random-effects model for IVW was adopted in this study. MR-PRESSO identified some significant outliers, and after removing these outliers, the results were consistent with previous analyses. “Leave-one-out” sensitivity analysis affirmed the above results. The funnel plot suggested a symmetrical distribution of SNP loci, indicating that the result is not susceptible to potential bias (Supplementary Figure).

## Discussion

Based on the 2-sample MR analysis, our results showed that TMAO and carnitine were associated with systolic BP. Each 1 unit increase in TMAO and carnitine was associated with an increase in systolic BP. However, there was not statistically significance of TMAO/carnitine for systolic BP in MR Egger, indicating that our results may be affected by potential deviation. In addition, we also found no evidence of causal effects of betaine and choline on BP.

TMAO, which can be converted from trimethylamine (TMA), is a metabolite of choline, lecithin, and carnitine (5, 10). Dietary choline and L-carnitine in the intestine produce TMA under the action of multiple enzymes. TMA is absorbed in the intestinal epithelium and transported to the liver, where it is further oxidized by flavin-containing monooxygenase 3 to form TMAO (5, 6). In this process, TMAO and its precursors have been reported to be closely associated with BP in numerous observational studies (25). For example, several experimental studies have elaborated a close relationship between TMAO and BP (5, 7), and previous cohort studies have also found a higher rate of hypertension in patients with high TMAO concentrations (26, 27). These findings were confirmed by a further systematic review that included 8 cohort studies with a total of 11,750 healthy controls and 6,176 hypertensive patients (8), which showed that high levels of TMAO were linked to a higher prevalence of hypertension compared with low levels of TMAO (RR: 1.12; 95% CI: 1.06–1.17). Each 5 μmol/L increase in TMAO level was associated with a 9% increase in the risk of hypertension (RR: 1.09; 95% CI: 1.05, 1.14), and each 10 μmol/L increase in TMAO level was linked to a 20% increased risk of hypertension (RR: 1.20; 95% CI: 1.11, 1.30; *P* < 0.0001) (8). In addition, further studies have also suggested that lowering blood TMAO concentrations can reduce BP levels (28–30). For instance, in a controlled study, probiotics improved BP levels in patients by lowering TMAO (28), and some other drugs, such as minocycline and enalapril, have been shown to regulate BP by mediating TMAO in clinical trials (29, 30). Overall, the results of these studies are similar to ours, suggesting a causal effect of TMAO on BP.

Additional studies have also found a relationship between precursors of TMAO such as carnitine, choline, and betaine and BP (9–12). However, there are apparent conflicting results from these studies. In a cross-sectional survey, physiological carnitine level was positively associated with increased BP in African and Caucasian men, which is in keeping with OmniHeart's and our MR findings (31), indicating that carnitine can affect systolic but not diastolic BP. Nevertheless, in a meta-analysis including 10 eligible RCTs containing 427 cases and 424 control populations (32), the authors found that carnitine supplementation reduced diastolic BP levels. Taken together, these results suggested that endogenous carnitine and exogenous carnitine supplementation may have different effects on BP. However, the specific mechanism remains obscure.

The mechanism by which TMAO and its precursors affect BP is currently unknown. Limited evidence has demonstrated that TMAO infusion can prolong the effects of hypertension in a model of angiotensin II-induced hypertension (33), suggesting that TMAO may enhance susceptibility to hypertension. In a further study, Brunt et al. (34) demonstrated that TMAO could promote the production of advanced glycation end products and superoxide-stimulated oxidative stress, thus inducing atherosclerosis and increasing systolic BP. In fact, a large number of studies have focused on TMAO promoting the formation of arteriosclerosis. Taking into account the inextricable link between atherosclerosis and hypertension, the following mechanisms should be considered (35–38). Firstly, TMAO can upregulate the number of scavenger receptors on the surface of macrophages and promote foam cell formation. Secondly, TMAO can affect the metabolism of cholesterol and lipoproteins by interfering with the reverse transport of cholesterol from extrahepatic organs and tissues into the liver. Thirdly, TMAO suppressed the expression of bile acid synthesis enzymes and reduced the rate of cholesterol metabolism. Fourthly, high levels of plasma TMAO may elevate the expression of cytokines such as interleukin-1β, interleukin-18, and tumor necrosis factor-α, while decreasing the expression of anti-inflammatory cytokines such as interleukin-10, which further promote atherogenesis and ultimately lead to hypertension.

This is the first MR study to explore the effect of TMAO and its precursors on BP. Our study has some advantages. For example, data on intestinal metabolites and BP in the study were obtained from the largest sample in the GWAS study; besides, in order to explain the potential factors, four MR methods (MR-PRESSO), Weighted median, MR Egger and Maximum likelihood were conducted for sensitivity analysis of IVW result. However, our study also has some weaknesses. First, this study used pooled data from the GWAS studies and could not evaluate the non-linear relationship between TMAO and BP, and also, due to the limitations of data, we were unable to perform subgroup analyses. Second, the inclusion of populations of European ancestry in this study may limit the application of the results to other ethnic people. Thirdly, since this is a cross-sectional design, it may have some impact on the reliability of our results. Fourthly, MR Egger reported that there was not statistically significance of TMAO/carnitine for systolic BP, indicating that our results may be affected by potential deviation. Fifthly, because we did not have access to raw data, therefore, we could only estimate the causality of the two, and at the same time, we could not conclude a detailed causal relationship of TMAO and the high systolic BP. Finally, we can only make a preliminary judgment about this causality of TMAO and carnitine with systolic BP by the MR approach, and the underlying biological mechanisms of the two are still not fully clarified. Therefore, more studies were still needed to prove the relationship between them.

In summary, we found a positive causal relationship of TMAO and carnitine with systolic BP, which was also confirmed by several previous studies. Further investigation of the mechanism between the two is suggested, and in addition, trials of targeted agents against TMAO need to be carried out.

## Data availability statement

The datasets presented in this study can be found in online repositories. The names of the repository/repositories and accession number(s) can be found in the article/supplementary materials.

## Ethics statement

Ethical review and approval was not required for the study on human participants in accordance with the local legislation and institutional requirements. The patients/participants provided their written informed consent to participate in this study. Written informed consent was obtained from the individual(s) for the publication of any potentially identifiable images or data included in this article.

## Author contributions

Study design and drafting manuscript: HW and ZZ. Data collection and data analysis: HW and QL. Data interpretation: XD and LC. All authors take responsibility for the integrity of the data analysis and participated and approved the final version of manuscript.

## Funding

This work was supported by National Natural Science Foundation of China [Grant 81300243], Sichuan Administration of Traditional Chinese Medicine [2020JC0010], Chengdu Health Commission Medical Research Project [2021206], Project of Sichuan Science and Technology Department [19YYJC0580]. Chengdu High-level Key Clinical Specialty Construction Project. Chengdu Science and Technology Bureau Technology Innovation Project [2019-YF05-00523-SN].

## Conflict of interest

The authors declare that the research was conducted in the absence of any commercial or financial relationships that could be construed as a potential conflict of interest.

## Publisher's note

All claims expressed in this article are solely those of the authors and do not necessarily represent those of their affiliated organizations, or those of the publisher, the editors and the reviewers. Any product that may be evaluated in this article, or claim that may be made by its manufacturer, is not guaranteed or endorsed by the publisher.
