# Supplementary material for: Trimethylamine N-oxide and its precursors in relation to blood pressure: A mendelian randomization study
Source: Front Cardiovasc Med. 2022 Jul 22;9:922441. doi: 10.3389/fcvm.2022.922441 (PMC9354484; doi:10.3389/fcvm.2022.922441)
Supplement: Supplementary file 2 [file Data_Sheet_1.pdf]

Supplementary Information

Trimethylamine N-oxide and Its Precursors in relation to Blood Pressure: A Mendelian Randomization Study

Han Wang, Qiang Luo, Xunshi Ding, Lifang Chen, Zheng Zhang

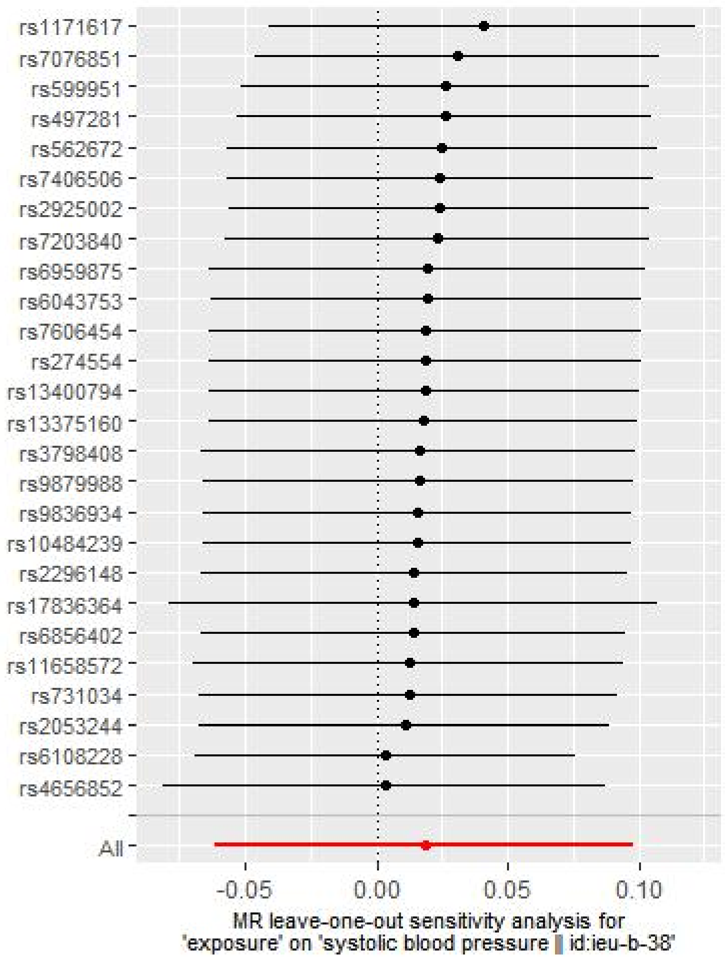

Figure 1: Leave-one-out plot to visualize causal effect of betaine on the risk of systolic blood pressure when leaving one SNP out.

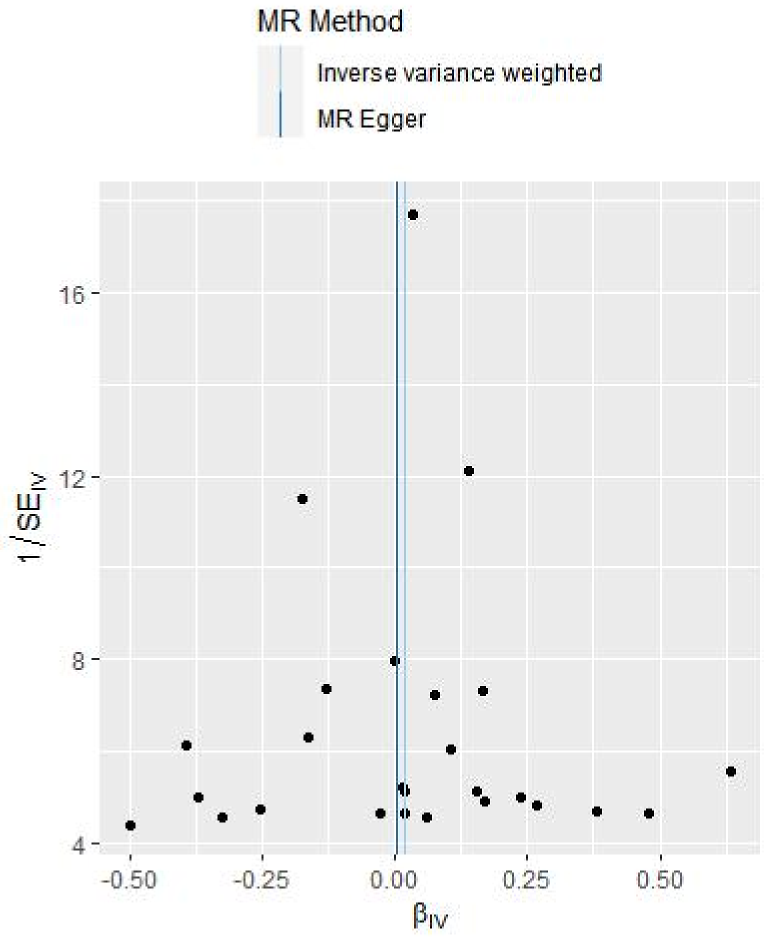

Figure 2: Funnel plots to visualize overall heterogeneity of Mendelian randomization (MR) estimates

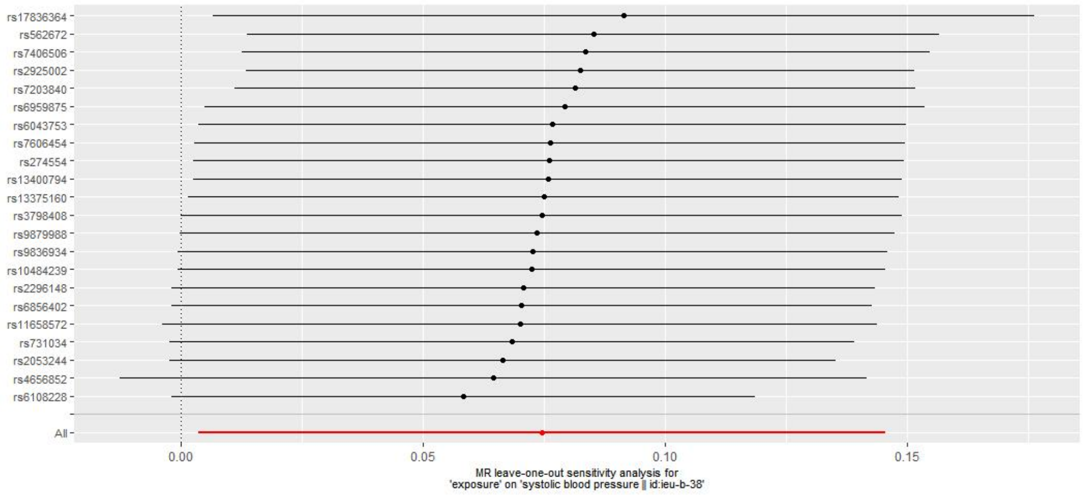

Figure 3: Leave-one-out plot to visualize causal effect of carnitine on the risk of systolic blood pressure when leaving one SNP out.

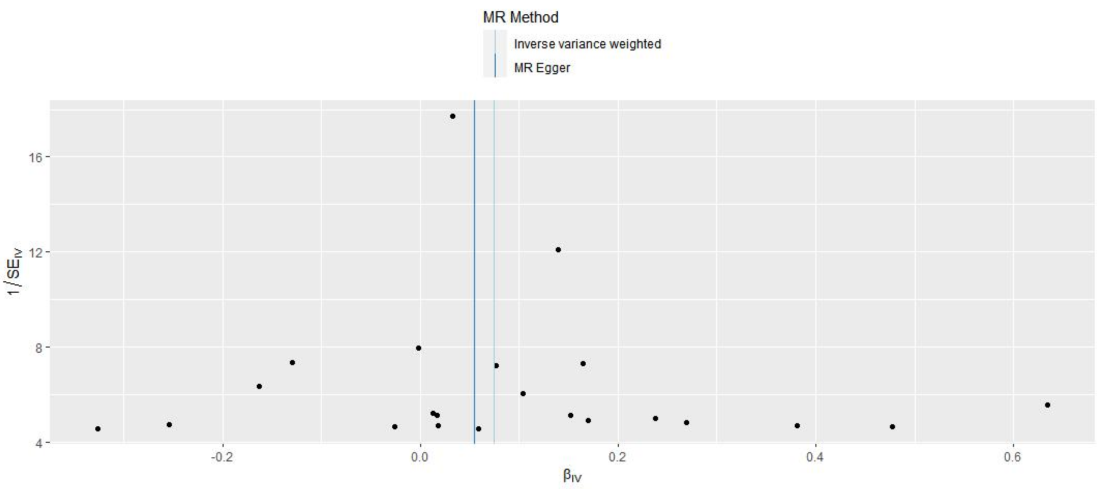

Figure 4: Funnel plots to visualize overall heterogeneity of Mendelian randomization (MR) estimates

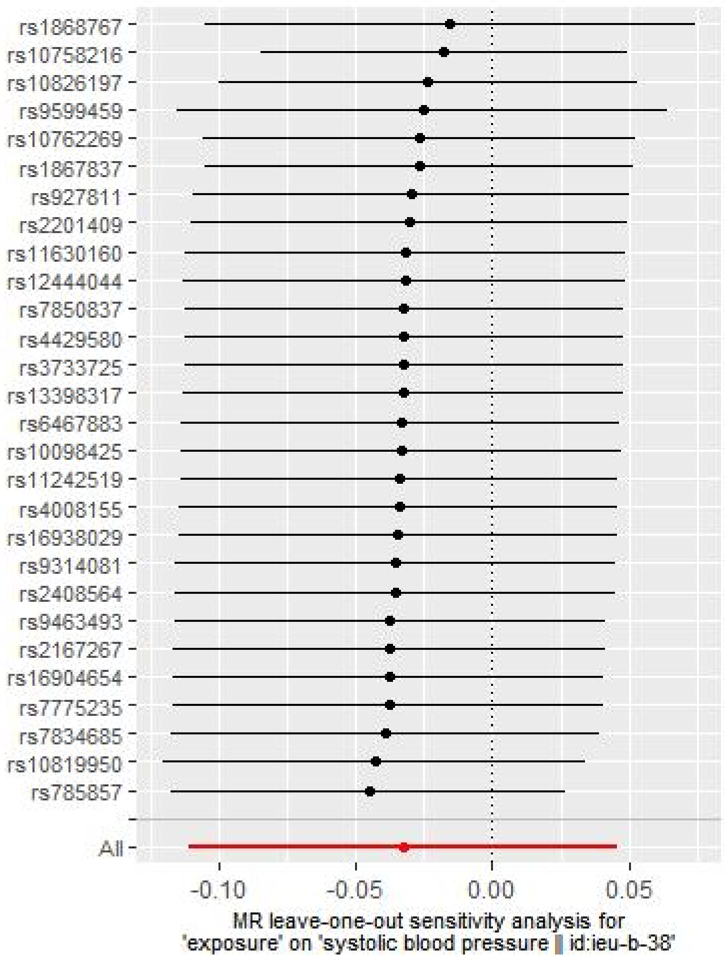

Figure 5: Leave-one-out plot to visualize causal effect of choline on the risk of systolic blood pressure when leaving one SNP out.

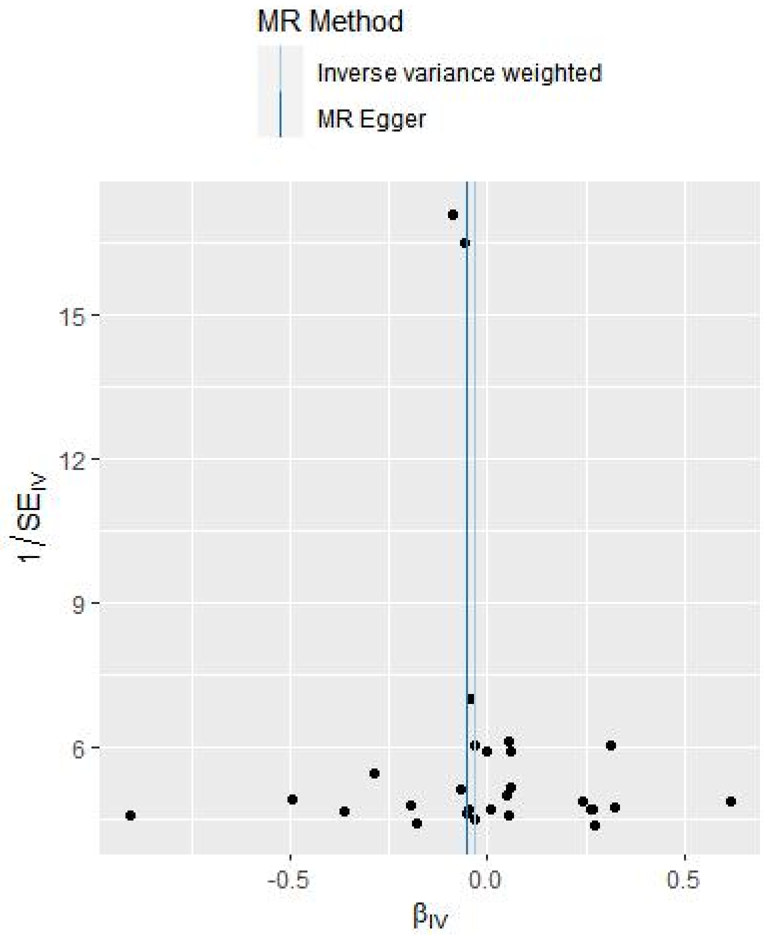

Figure 6: Funnel plots to visualize overall heterogeneity of Mendelian randomization (MR) estimates

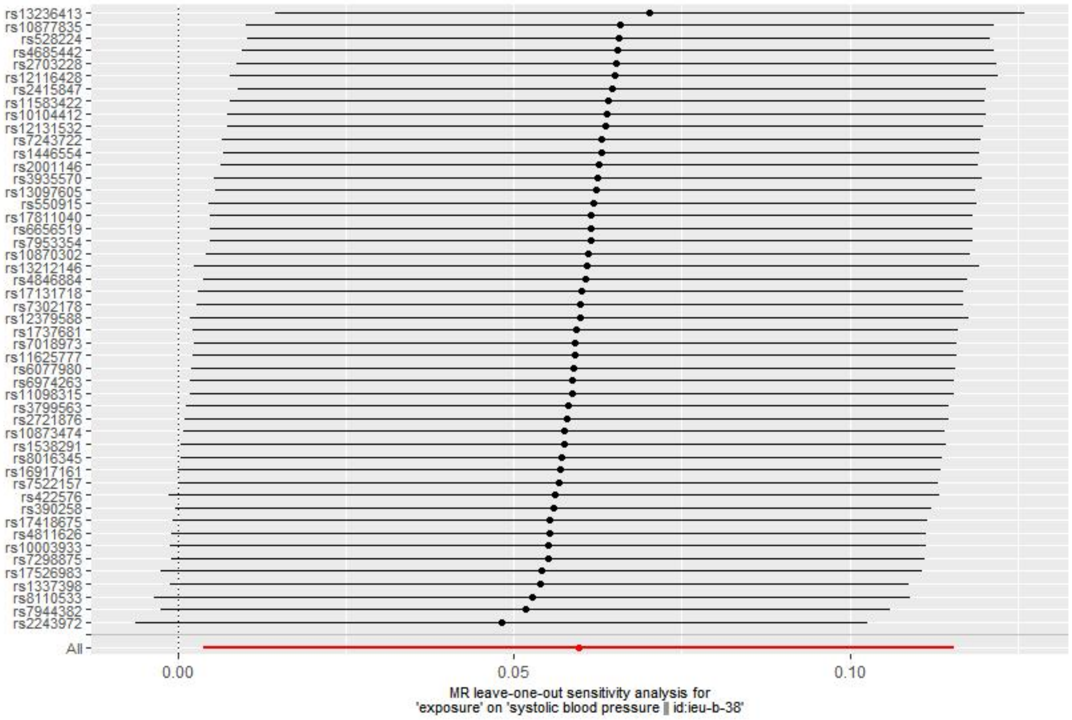

Figure 7: Leave-one-out plot to visualize causal effect of trimethylamine_N_oxide on the risk of systolic blood pressure when leaving one SNP out.

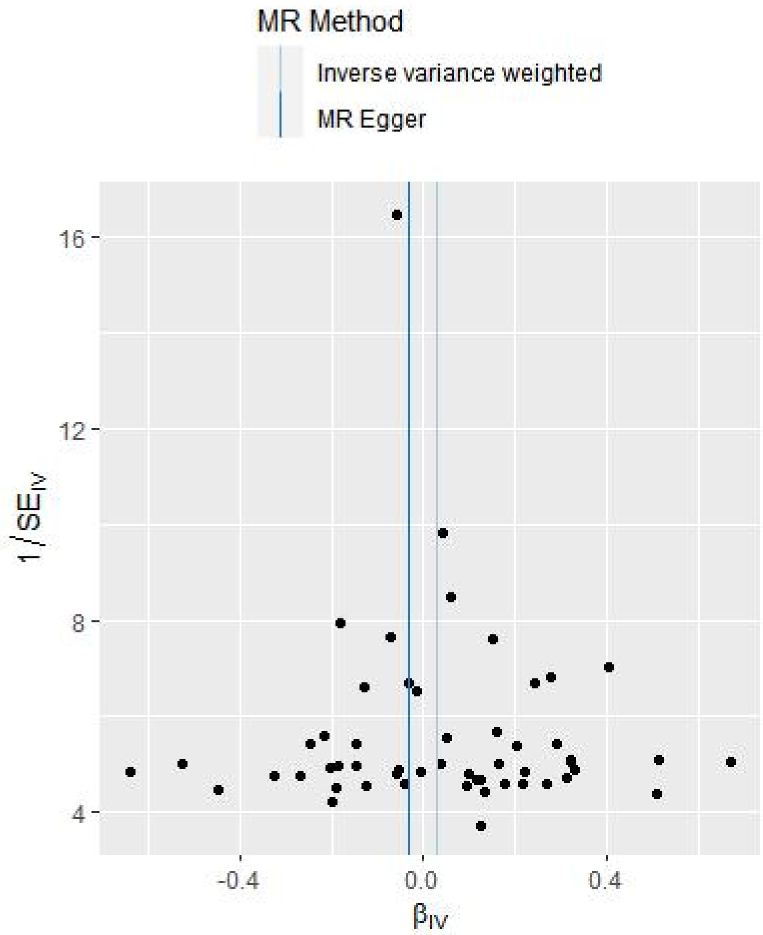

Figure 8: Funnel plots to visualize overall heterogeneity of Mendelian randomization (MR) estimates

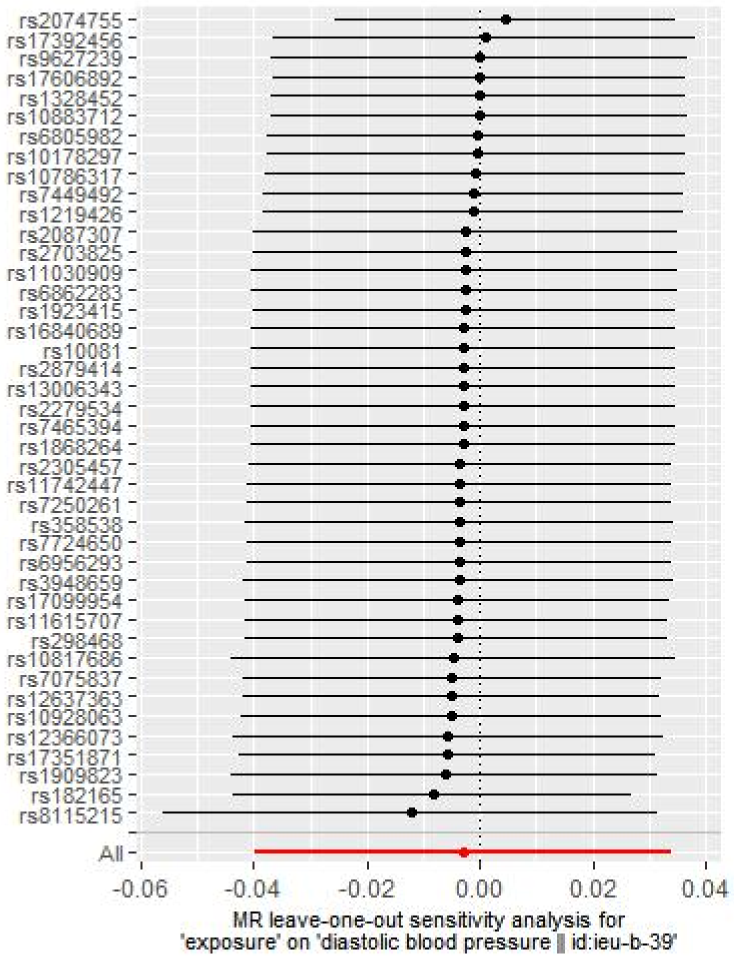

Figure 9: Leave-one-out plot to visualize causal effect of betaine on the risk of diastolic blood pressure when leaving one SNP out.

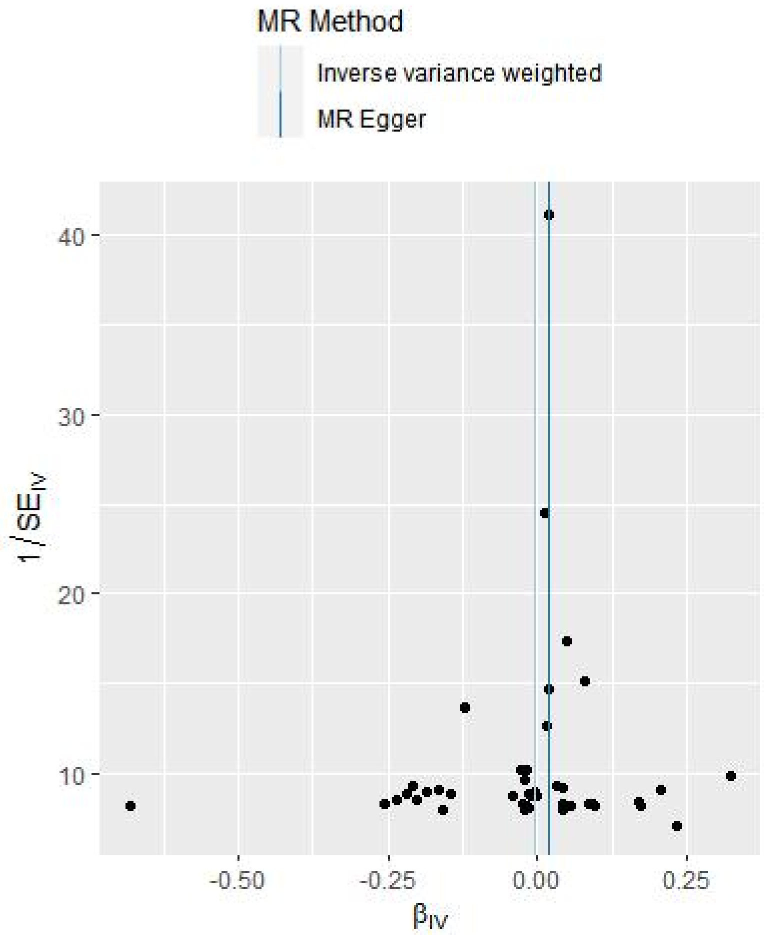

Figure 10: Funnel plots to visualize overall heterogeneity of Mendelian randomization (MR) estimates

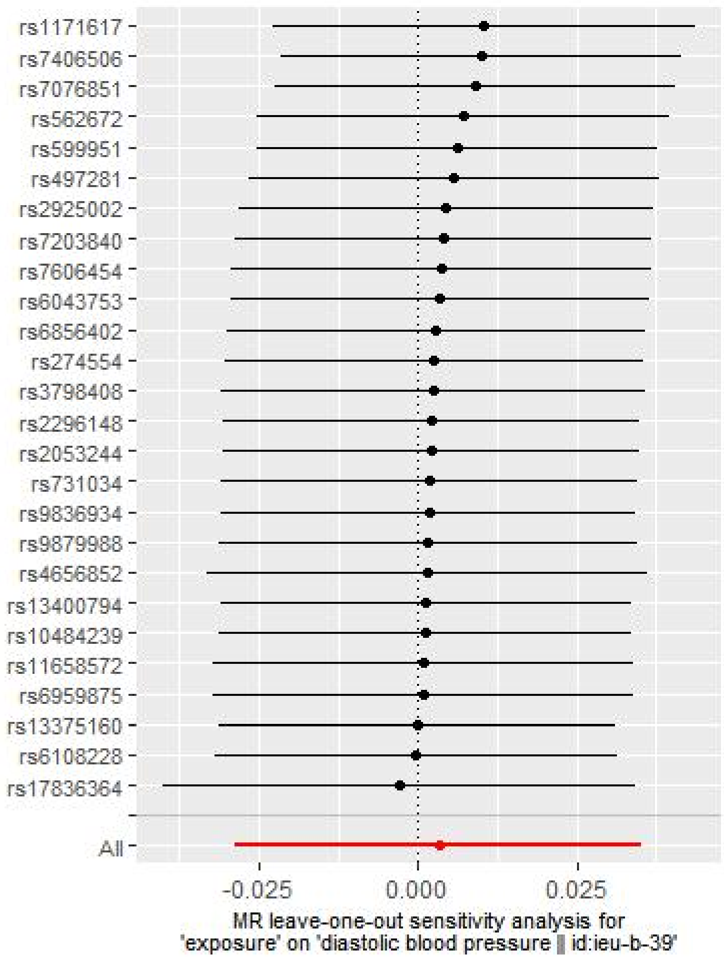

Figure 11: Leave-one-out plot to visualize causal effect of carnitine on the risk of diastolic blood pressure when leaving one SNP out.

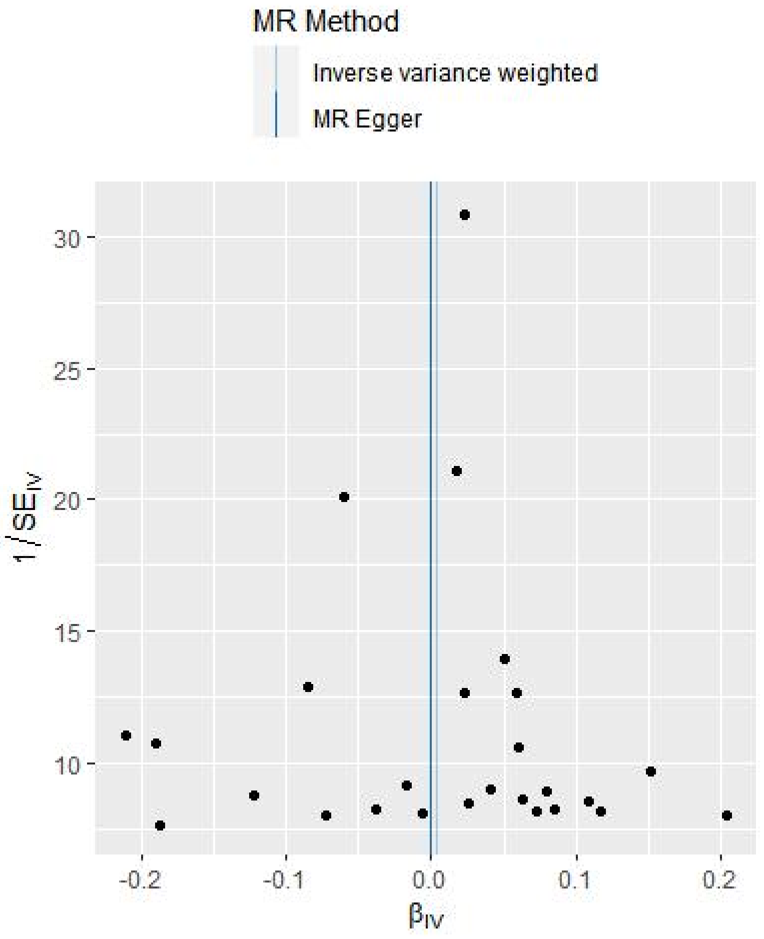

Figure 12: Funnel plots to visualize overall heterogeneity of Mendelian randomization (MR) estimates

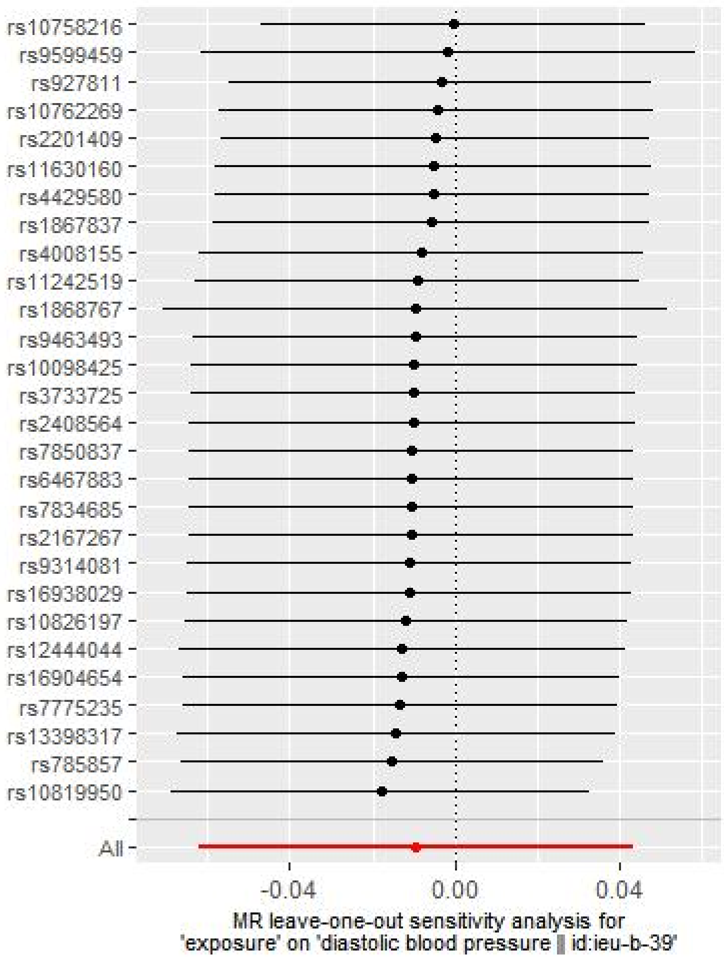

Figure 13: Leave-one-out plot to visualize causal effect of choline on the risk of diastolic blood pressure when leaving one SNP out.

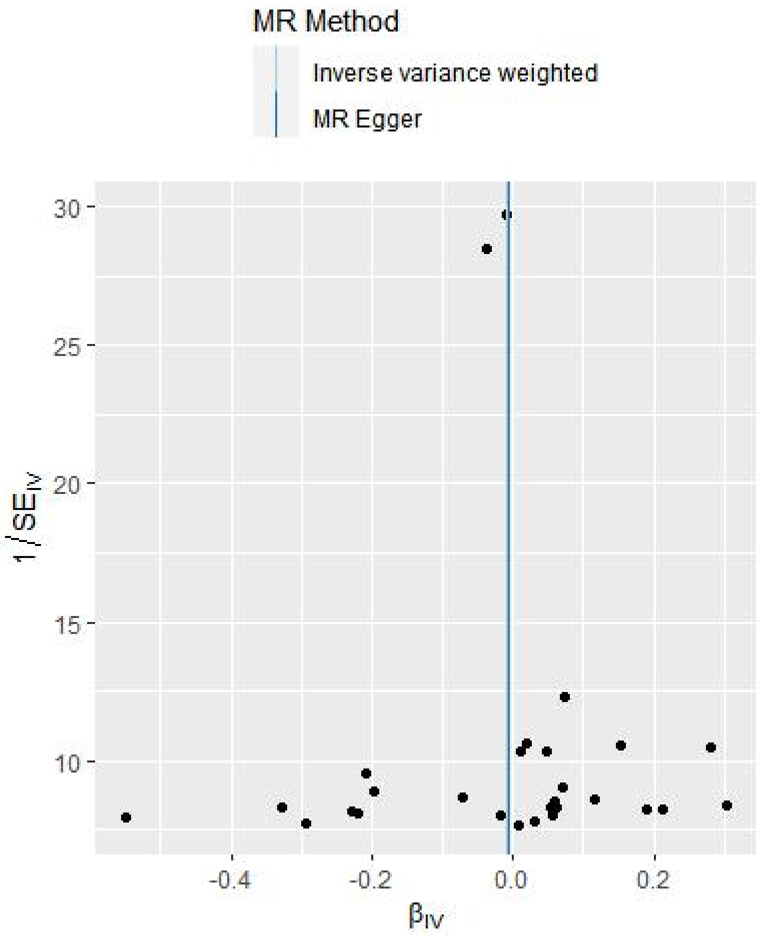

Figure 14: Funnel plots to visualize overall heterogeneity of Mendelian randomization (MR) estimates

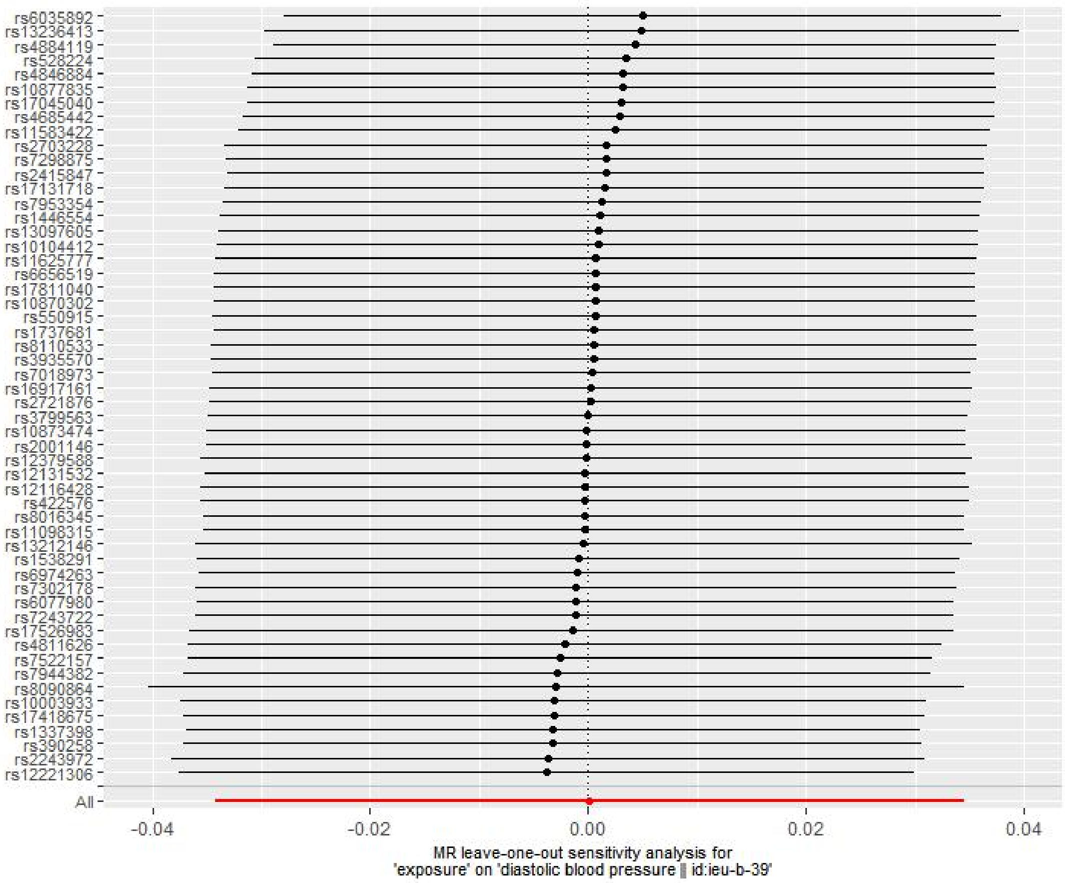

Figure 15: Leave-one-out plot to visualize causal effect of trimethylamine_N_oxide on the risk of diastolic blood pressure when leaving one SNP out.

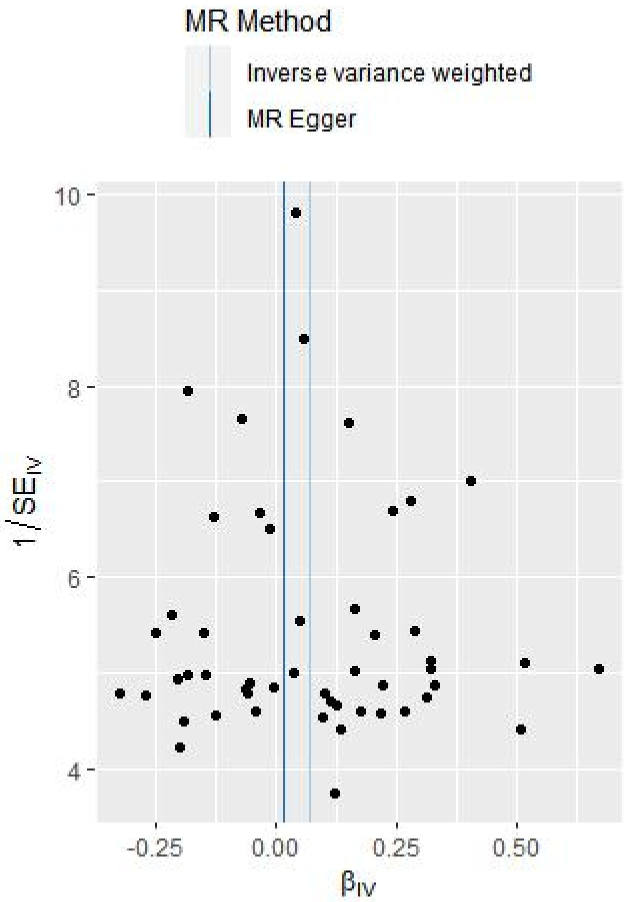

Figure 16: Funnel plots to visualize overall heterogeneity of Mendelian randomization (MR) estimates
